# Optimized Aptamer-Conjugated Gold Nanoparticles for Specific Detection of GII.4 Human Norovirus in Feces

**DOI:** 10.3390/bios15110713

**Published:** 2025-10-28

**Authors:** Chao Cheng, Xiaomeng Zhang, Gaoyang Li, Minjia Sun, Wenjing Zheng, Jingjing Li, Jing Liu, Xuanyi Wang, Youhua Xie, Shouhong Xu, Junqi Zhang

**Affiliations:** 1 MOE/NHC/CAMS Key Laboratory of Medical Molecular Virology, Shanghai Institute of Infectious Disease and Biosecurity, School of Basic Medical Sciences, Fudan University, Shanghai 200032, China; 23211010048@m.fudan.edu.cn (C.C.); 24211010055@m.fudan.edu.cn (X.Z.); 17301020061@fudan.edu.cn (J.L.); liujing212@fudan.edu.cn (J.L.); yhxie@fudan.edu.cn (Y.X.); 2 Key Laboratory for Advanced Materials, School of Chemistry and Molecular Engineering, East China University of Science and Technology, Shanghai 200237, China; Y20210037@mail.ecust.edu.cn (G.L.); sunmj@conbapharm.com (M.S.); 3 Zhejiang CONBA Pharmaceutical Co., Ltd., Hangzhou 310052, China; 4 Key Laboratory of Medical Molecular Virology of MoE & MoH, Institutes of Biomedical Sciences, Fudan University, Shanghai 200032, China; 22111510073@m.fudan.edu.cn (W.Z.); xywang@shmu.edu.cn (X.W.)

**Keywords:** aptamer, human norovirus, gold nanoparticles, colorimetric assay

## Abstract

Human norovirus (HuNoV), particularly the GII.4 genotype, is a leading cause of acute gastroenteritis worldwide, posing a significant public health and economic burden due to its low infectious dose. To address the need for rapid and sensitive detection, we developed a colorimetric biosensor utilizing a structure-optimized aptamer and gold nanoparticles (AuNPs). Biotin-modified aptamers could protect AuNPs from aggregation in salt solution. Upon specific binding to GII.4 HuNoV virus-like particles (VLPs), this protective effect is disrupted, leading to AuNP aggregation and a measurable color shift quantified by the A620/A520 absorbance ratio. Under optimized conditions, the assay demonstrated a linear response (y = 0.004597x + 0.3277, R^2^ = 0.9922) to GII.4 HuNoV VLP concentrations ranging from 0.1 to 3.0 μg/mL, with the recovery rates between 91.74% and 106.43%. The biosensor exhibited high specificity for GII.4 HuNoV, showing minimal cross-reactivity with other common diarrheal pathogens, and achieved an exceptional detection limit of 27.2 copies/mL in a fecal matrix. Molecular docking and point mutation confirmed the critical roles of specific nucleotide bases (T20, C22, G31, and G44) in the aptamer and the Asn55 residue in the viral capsid for binding. This work establishes a sensitive, rapid, and cost-effective aptamer-based colorimetric platform suitable for the large-scale monitoring of GII.4 HuNoV.

## 1. Introduction

Human norovirus (HuNoV) is a pathogen responsible for non-bacterial acute gastroenteritis in humans and was first identified via immunoelectron microscopy in 1972 [1]. HuNoV is highly contagious (with a 50% infectious dose between 18 and 2800 gene copies [2]) and environmentally stable (can survive on surfaces of objects for up to 2 weeks and in the environment for up to 9 weeks after outbreak [3]). These attributes contribute to an estimated 700 million cases of diarrhea and approximately USD 4 billion in medical costs annually worldwide [4]. Given that the infectious dose can be as low as 100 viral copies [5], accurate and sensitive detection of HuNoV in samples is essential.

Current detection methods for HuNoV include electron microscopy, real-time reverse transcription quantitative polymerase chain reaction (RT-qPCR) targeting the ORF1/ORF2 junction of the HuNoV genome [6], and antibody-based enzyme-linked immunoassays [7]. Among them, electrical observation and RT-qPCR require sophisticated instrumentation and skilled personnel, making them unsuitable for rapid, high-throughput, or on-site testing. An enzyme-linked immunoassay has good portability, but the antibodies used in this method are relatively expensive, differential between batches, and require stringent storage and transportation conditions, limiting its widespread application [8]. Therefore, there is a need to develop simpler, faster, and more cost-effective methods for detecting HuNoV in outbreak samples.

Nucleic acid aptamers are a class of single-stranded DNA or RNA selected from synthetic random oligonucleotide libraries through systematic evolution of ligands by exponential enrichment (SELEX) [9]. As a chemical antibody, they bind specifically to target molecules and have the advantages of easy preparation and labeling modification, good stability, and low price [10]. Therefore, aptamers against various antibiotics, toxins, and small molecules have been screened and used in diagnostics [11,12,13,14,15]. Current aptamer-based detection strategies include fluorescence assays [16], electrochemical biosensor [17], gel migration [18], and isothermal titration calorimetry [19]. These methods generally require specialized instrumentation and high level of technical skills, thus limiting their application.

Nanomaterials are widely used as drug delivery vehicles and sensing platforms [20]. Among them, gold nanoparticles (AuNPs) are one of the most popular tools for designing optical analytical devices due to their unique optical and electrical properties, high chemical stability, and simple synthesis [21]. AuNPs undergo a visible color shift from red to blue-purple upon aggregation in salt solution, enabling their use as sensitive probes for detecting chemical and biological analytes [22], which can be distinguished directly visually [23]. Combining the affinity and specificity of aptamers and colorimetric properties of AuNPs, efficient sensors for different targets can be developed [24]. Specifically, AuNPs aggregate in high-salt environments such as NaCl solution, resulting in a distinct color change [25]. Aptamers adsorbed on the AuNP surface via electrostatic or coordinate bonding can protect the nanoparticles from salt-induced aggregation [26]. In the presence of a target protein, the aptamer preferentially binds to the target through hydrogen bonding, van der Waals interactions, or electrostatic forces [27], leading to desorption from the AuNP surface. The unprotected AuNPs then aggregate in NaCl solution, producing a measurable colorimetric signal. The method is simple, cheap, and easy to use, making it suitable for applications in environmental monitoring, public health, and clinical diagnostics [11,12,13,14,15,28].

In this study, we optimized a previously reported aptamer specific to GII.4 HuNoV virus-like particles (VLPs) [29] and gained new aptamers with significantly enhanced affinity and specificity. Using the optimal aptamer as a biorecognition element immobilized on AuNPs, we established a visual detection method for GII.4 HuNoV. The assay successfully detected both GII.4 HuNoV VLPs—used as a safe experimental surrogate—and GII.4 HuNoV in clinical fecal samples. Our work provides a real-time, convenient, and cost-effective platform for large-scale screening of GII.4 HuNoV. Furthermore, we elucidated the binding mechanism between the aptamer and the GII.4 HuNoV VP1 capsid protein, providing a foundation for future applications of this aptamer.

## 2. Materials and Methods

### 2.1. Reagents and Instrumentation

All the aptamers were synthesized by Tsingke Biotech Co., Ltd. (Shanghai, China). The water used for all experiments was purified by Milli-Q system (Millipore, Bedford, MA, USA). Ultraviolet–visible absorption spectra were recorded on a Microplate Absorbance Spectrophotometer (Bio-Rad, Hercules, CA, USA). Gold nanoparticles with an average diameter of 15 nm were purchased from www.biotyscience.com (Cat No: ABZW-1-15, Beijing, China). Table S1 details various HuNoV-related targets (GII.4 HuNoV VLP, GII.4 HuNoV, and GII.4 HuNoV VP1) used in this study and their corresponding experiments.

### 2.2. Preparation of Norovirus VLP

HuNoV GII.4 strain Hu/GII.4/DBM15-156/2015 (GenBank ID: MG786781.1) and HuNoV GII.17 strain Hu/GII.P17_GII.17/KR/2015 (GenBank ID: NC_039475.1) were reported in our previous study [29]. We have also expressed a series of His-tagged HuNoV VLPs using a baculovirus expression system: HuNoV GI.3 strain (GenBank ID: MZ021600.1), HuNoV GII.2 strain Env/CHN/2016 (GenBank ID: NC_039476.1), HuNoV GII.3 strain Hu/US/1972 (GenBank ID: KY442319.1), and HuNoV GII.6 strain Hu/JP/2022 (GenBank ID: LC790056.1). To determine the expression and assembly of VLPs, we performed Sodium Dodecyl Sulfate Polyacrylamide Gel Electrophoresis (SDS-PAGE) and Western blotting (WB) with an anti-His monoclonal antibody. In addition, VLPs were negatively stained using 2% aqueous uranyl acetate, and then the morphology was observed by Transmission Electron Microscopy (TEM) (JEM-1400Flash, JEOL, Tokyo, Japan).

### 2.3. Enzyme-Linked Aptamer Sorbent Assay (ELASA)

The ELASA was similar to the previously described enzyme-linked immunosorbent assay method, except that the primary antibody binding the antigen is replaced by biotin-labeled aptamer, and the secondary antibody is anti-biotin antibody [30]. The experiment was conducted as previously published [29].

### 2.4. Optimization of Aptamer Concentration of the Colorimetric Assay

In order for the aptamers to modify the AuNPs, 40 μL of 3 μM aptamers, NH_2_-modified aptamers, or biotin-modified aptamers were added to 140 μL of colloidal gold solution, mixed, and incubated at 37 °C for 1 h, respectively. Then, 20 μL of 450 mM NaCl was added, and the wavelength ranging from 400 nm to 700 nm was recorded. To determine the lowest concentration of aptamer required to protect AuNPs from aggregation by NaCl, 40 μL of different concentrations of the biotin-AP 4-11 was added to 140 μL of colloidal gold solution and mixed, and we performed the same experiment as above.

### 2.5. General Procedure for Detecting GII.4 HuNoV VLPs

The reactions were all carried out in 96-well enzyme labeling plates. Firstly, 100 μL of AuNPs was incubated with 40 μL of 2 μM biotin-AP4-11 at room temperature for 1 h. Then, 40 μL of GII.4 HuNoV VLPs or ddH_2_O was added to the solution and incubated at room temperature in the dark for 1 h, and 20 μL of 450 mM NaCl solution was added. The solution was allowed to stand in the dark for 10 min, and the absorbance values at 520 nm and 620 nm were measured. To obtain the fitting curve, GII.4 HuNoV VLPs with concentration gradients were selected. A standard curve was generated using GraphPad Prism 6.01 software.

### 2.6. Specificity and Broad Spectrum of the Colorimetric Assay

The concentrations of GII.4 HuNoV VLPs, *E. coli* lysate, EV71, astrovirus, and coronavirus S protein used in the specificity experiment of the colorimetric assay were all 5 μg/mL, and ddH_2_O was used for the blank control. The experimental conditions were kept consistent. The concentration of each genotype of HuNoV VLPs used in the broad-spectrum experiments was 5 μg/mL.

### 2.7. Detection of GII.4 HuNoV in Real Sample

Clinical HuNoV samples of each genotypic subtype were generously gifted by Prof. Xuanyi Wang of Fudan University and classified into genotypic subtypes in a previous work [31]. The fecal samples were divided into two parts. One copy was used for the extraction of viral RNA and quantification of gene copy number using a norovirus nucleic acid standard reagent kit (Meizheng Biotechnology, Rizhao, China). Based on the quantification results, the other sample was diluted, and the copy numbers of different genotypes in the same volume were the same. The samples were diluted so that the concentration of viral copies was consistent across genotype subtypes.

### 2.8. Recovery of GII.4 HuNoV VLPs in Fecal Sample

GII.4 HuNoV VLPs were determined by the standard addition and recovery experiment. Validated HuNoV negative stool samples were diluted 100-fold using ddH_2_O and supplemented with a range of concentrations of GII.4 HuNoV VLPs. Then, the samples were analyzed with the above colorimetric assay. The recovery was calculated as follows: recovery rates = (concentration/spiked concentration) × 100%.

### 2.9. Aptamer Structure Modification and Simulated Docking with GII.4 HuNoV VP1

Secondary structure folding analysis and ∆G prediction of candidate aptamers were carried out using the DNA Mfold online server (http://mfold.rna.albany.edu/, accessed on 23 November 2024) [32]. The tertiary structure of the aptamer was predicted by the RNA Composer server (http://rnacomposer.ibch.poznan.pl/, accessed on 5 December 2024), and the resulting three-dimensional RNA-generated PDB files were manually converted to DNA format in MOE (2022) software. The three-dimensional structure of GII.4 HuNoV VP1 was predicted by the SWISS-MODEL server (https://swissmodel.expasy.org, accessed on 5 December 2024). The obtained aptamer PDB files were then molecularly docked with the GII.4 HuNoV VP1 PDB files in ZDOCK server (https://zdock.wenglab.org/, accessed on 7 December 2024).

## 3. Results and Discussion

### 3.1. Principle of Colorimetric Detection

**Figure 1:**
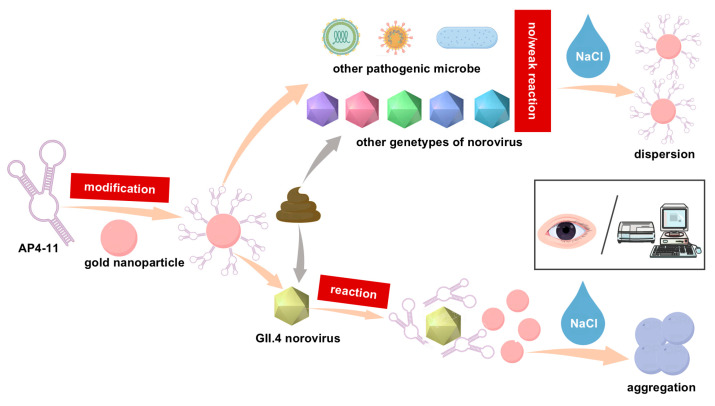
Schematic illustration of colorimetric assay of GII.4 HuNoV.

Figure 1 illustrates the principle for detecting GII.4 HuNoV using aptamer-modified AuNPs. In the absence of GII.4 HuNoV, the aptamer remains in a random coil conformation. The exposed positively charged bases or modifying groups interact electrostatically with the negatively charged AuNPs and adsorb onto their surface. Due to electrostatic repulsion and steric stabilization, the AuNPs remain highly stable and resist aggregation even in high concentrations of NaCl, retaining their original red color.

In contrast, when the sample contains GII.4 HuNoV, it binds to the aptamer with higher affinity through electrostatic interactions, hydrogen bonding, spatial complementarity, and other effects [33]. This binding induces a conformational change in the aptamer from a random coil to a folded, rigid secondary structure, which prevents its adsorption onto the AuNP surface. As a result, the AuNPs become unprotected. The subsequent addition of NaCl neutralizes the surface charge of the AuNPs, leading to their aggregation and a visible color change from red to purple-gray.

The absorbance of the solution at 520 nm and 620 nm can be measured using a microplate spectrophotometer, and quantitative analysis can be performed based on the A620/A520 ratio.

### 3.2. Aptamer Modification and Characterization

**Figure 2:**
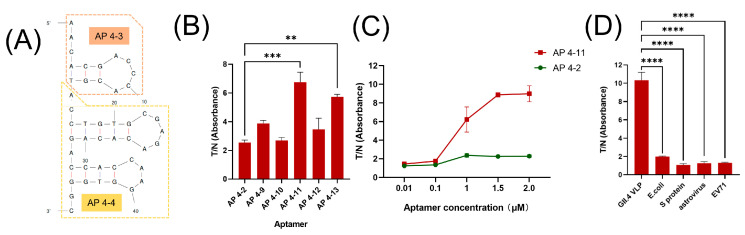
(**A**) Secondary structure prediction of AP4-11 (different colors indicate the source aptamer of the sequence). (**B**) Results of ELASA performed with optimized aptamers of AP4-2 (GII.4 HuNoV VLP concentration: 3 μg/mL; aptamer concentration: 1 μM). (**C**) Results of ELASA performed with different aptamer concentrations of AP4-11. (**D**) Comparison of AP4-11 to GII.4 HuNoV VLPs versus unrelated viruses/proteins using ELASA. The data are reported as means ± SEMs of triplicate wells. ****, *p* < 0.0001, ***, *p* < 0.001, and **, *p* < 0.01.

We performed sequence modification and structural prediction on previously screened aptamers against GII.4 HuNoV VLP [29]. As summarized in Table S2, four original aptamers were selected for rational redesign. Modifications involved retaining stem–loop structures potentially critical for aptamer–receptor binding, removing unpaired bases, or concatenating stem–loop motifs in tandem. This process yielded five new aptamers ranging from 38 to 47 bases in length, with predicted ΔG values between −6.06 and −15.49 kcal/mol. A lower ΔG generally indicates greater structural stability. The predicted secondary structures (Figure 2A and Figure S1) reveal that each aptamer contains two to three stem–loop motifs.

To evaluate whether the modifications enhanced target affinity, we performed ELASA experiments for AP4-2 (the aptamer with the strongest affinity obtained from the screening previously verified) with the modified aptamers, respectively. As shown in Figure 2B, both AP4-11 and AP4-13 exhibited significantly stronger affinity for GII.4 HuNoV VLP compared to AP4-2. We further characterized AP4-11 by testing its binding across a range of aptamer concentrations. While AP4-11 and AP4-2 showed similar affinities at low concentrations (0.01–0.1 μM), AP4-11 demonstrated markedly stronger binding at higher concentrations (1.0–2.0 μM) (Figure 2C). Moreover, when AP4-2 reached saturation at 1.0 μM, the T/N value for AP4-11 continued to increase with concentration and did not plateau until 1.5 μM, indicating a higher binding capacity of AP4-11 for GII.4 HuNoV VLPs.

Since structural optimization may alter specificity [34], we also assessed AP4-11 against other common diarrheal pathogens. As illustrated in Figure 2D, AP4-11 maintained high specificity and showed no cross-reactivity. In summary, through rational sequence modification of previously reported aptamers, we obtained a novel aptamer, AP4-11, with enhanced affinity and high specificity for GII.4 HuNoV VLPs.

### 3.3. Characterization of AuNPs

As shown in Figure S2, the prepared AuNPs are red in color and are spherical, uniform, and well dispersed under TEM, with an average diameter of approximately 15 nm. The UV–Vis spectrum showed a characteristic absorption peak at 520 nm (Figure S2A,B). Upon the addition of 2 M NaCl solution, the AuNPs aggregated, resulting in a color change from red to purple-gray. Correspondingly, the absorption peak at 520 nm decreased, while the absorbance in the 600–700 nm range increased (Figure S2C,D).

### 3.4. Optimization of NaCl and Aptamer Concentrations

To determine the optimal salt concentration for subsequent colorimetric assays, we measured the color change and absorbance spectra (400–700 nm) of AuNPs in the presence of different NaCl concentrations. Based on the results, 450 mM NaCl was selected for all further experiments (Figure S3A,B).

**Figure 3:**
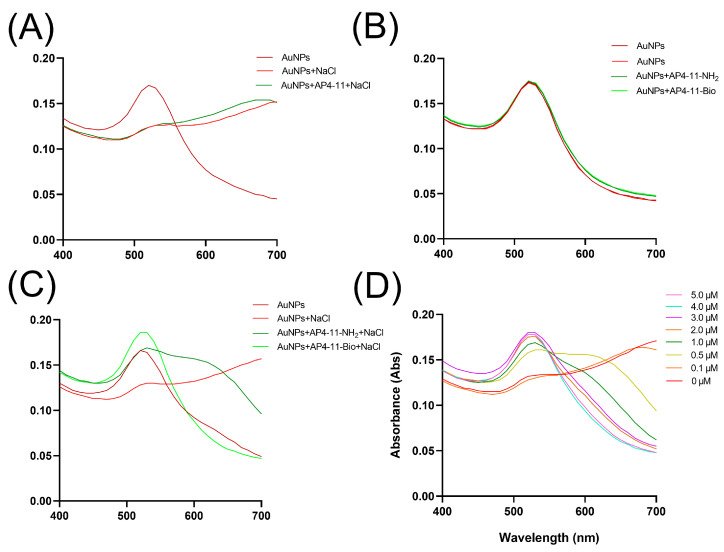
Optimizations of colorimetric conditions (aptamer modification and concentration). (**A**) Protective effect of AP4-11 on AuNPs. (**B**) Absorbance of AuNPs and AuNPs incubated with NH_2_- or biotin-modified AP4-11. (**C**) Protective effect of NH_2_- or biotin-modified AP4-11 on AuNPs. (**D**) Relationship between absorbance and different concentrations of biotin-modified AP4-11.

We next optimized the aptamer concentration required to protect AuNPs from salt-induced aggregation. In contrast to some previous reports [35], initial experiments showed that adding 5.0 μM of unmodified AP4-11 failed to stabilize the AuNPs. As shown in Figure 3A, the AuNPs still aggregated upon NaCl addition, with absorbance spectra similar to the control (AuNPs + NaCl). We also attempted appending polyA tails to either the N- or C-terminus of AP4-11, but no protective effect was observed.

We next synthesized a 5′NH_2_-labeled aptamer, the type of labeling used in a previous study [14] to successfully modify AuNPs, and a 5′biotin-labeled aptamer. Incubation of AuNPs with 5.0 μM of either labeled aptamer did not alter the baseline absorbance spectrum (400–700 nm) (Figure 3B). However, after NaCl addition, both modified aptamers conferred protection against aggregation, with the 5′-biotin-labeled version performing more effectively. The absorbance profile of biotin-AP4-11-modified AuNPs closely resembled that of unstabilized AuNPs without NaCl, whereas the NH_2_-labeled aptamer led to a reduced peak at 520 nm and elevated absorbance at 620 nm (Figure 3C).

The difference in performance can be attributed to the distinct interaction mechanisms of each functional group with the AuNP surface [14,36,37]. The amino group forms a coordinate covalent bond with gold—a strong but single-point attachment—which may not sufficiently stabilize the aptamer’s secondary structure or mitigate electrostatic repulsion. In contrast, the sulfur atom in the tetrahydrothiophene ring of biotin exhibits high affinity for gold, and its hydrophobic valeric acid side chain facilitates additional van der Waals interactions with the gold surface. Furthermore, the ureido and carbonyl groups in biotin may participate in hydrogen bonding with surface-adsorbed water molecules or impurities, further enhancing stability.

Based on these results, we tested a range of concentrations of 5′-biotin-AP4-11 and found that 2.0 μM provided complete protection against NaCl-induced aggregation, with no observable changes in color or absorbance (Figure 3D). Therefore, 2 μM biotin-AP4-11 was selected for subsequent experiments.

### 3.5. Detection of GII.4 HuNoV VLPs with Aptasensor

**Figure 4:**
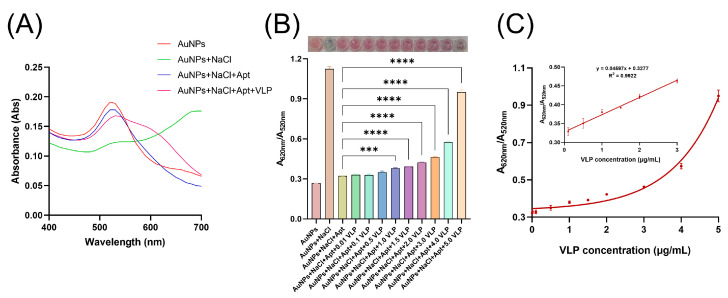
(**A**) Absorbance of different components in the colorimetric assay. (**B**) A620/A520 values obtained using different concentrations of GII.4 HuNoV VLPs in the colorimetric assay and corresponding images of color changes. (**C**) Quantitative analysis of GII.4 HuNoV VLPs. The data are reported as means ± SEMs. ****, *p* < 0.0001; ***, *p* < 0.001.

After optimizing the NaCl and aptamer concentrations for the colorimetric assay, we introduced GII.4 HuNoV VLPs into the system. As shown in Figure 4A, the presence of VLPs reduced the amount of aptamer bound to the AuNP surface. After NaCl addition, this resulted in a visible shift in the absorbance profile, characterized by a decrease at 520 nm and an increase at 620 nm.

To evaluate the sensitivity of the method, we tested different concentrations of GII.4 HuNoV VLPs. As the VLP concentration increased, the color of the AuNP solution transitioned progressively from red to purple and finally to purple-gray, accompanied by a gradual increase in the A620/A520 ratio (Figure 4B). A significant difference in A620/A520 was observed at 1 μg/m VLPs compared to the control (no VLPs). At 5 μg/mL VLPs, the solution color closely resembled that of unmodified AuNPs with NaCl alone. Based on the A620/A520 values, we estimated that VLPs competed for approximately 80% of the originally bound aptamer from the AuNP surface. In addition, as shown in Figure 4C, a strong linear correlation was observed between GII.4 HuNoV VLP concentration and A620/A520 within the range of 0.1–3 μg/mL following the equation y = 0.004597x + 0.3277 (R^2^ = 0.9922; x is the VLP concentration, and y is the value of A620/A520). In summary, this colorimetric assay effectively detects GII.4 HuNoV VLPs through both visual observation of color changes and spectrophotometric measurement of A620/A520, which provides support for large-scale rapid preliminary detection in the field.

### 3.6. Kinetic Analysis of the Colorimetric Assay

To determine the optimal reaction time, we monitored the time-dependent change in the A620/A520 ratio of the AuNP solution after the addition of NaCl. Measurements were taken at various time intervals following NaCl introduction. As shown in Figure S4, the A620/A520 ratio increased markedly within the first 10 min, reflecting rapid aggregation of unprotected AuNPs. After 10 min, the rate of increase slowed considerably, indicating that the aggregation process was largely complete and that the system was approaching equilibrium. In contrast, the A620/A520 ratio of the control group (AuNPs + Apt) in the absence of GII.4 HuNoV VLPs remained nearly constant throughout the observation period, confirming the stability of the colorimetric system under these conditions.

### 3.7. Specificity and Broad-Spectrum of the Colorimetric Assay

**Figure 5:**
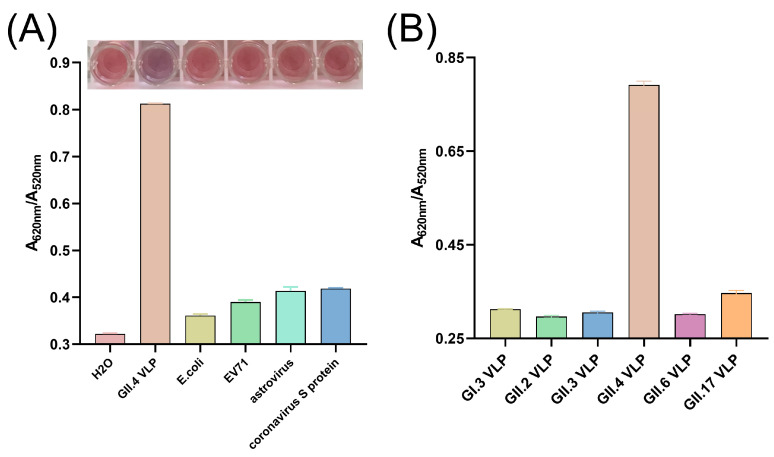
(**A**) Specificity of aptamer–AuNP colorimetric assay for the detection of GII.4 HuNoV VLPs. (**B**) Response of the colorimetric assay to various genotypes of HuNoV VLPs. The data are reported as means ± SEMs.

To assess the specificity of the colorimetric sensor for GII.4 HuNoV detection, we evaluated several potential interfering substances. Visual inspection revealed that only in the presence of GII.4 HuNoV VLPs did the AuNP solution change color from red to purple-gray, accompanied by an A620/A520 value of approximately 0.8. In contrast, solutions containing other pathogens remained red, consistent with the blank control (ddH_2_O), and exhibited A620/A520 values below 0.4 (Figure 5A).

To determine whether the colorimetric assay is applicable to other HuNoV genotypes, we also tested HuNoV VLPs from a panel of genetic subtypes, including GI.3 (representative of the GI genogroup), recently prevalent genotypes GII.2, GII.3, and GII.6, as well as GII.17, which is widespread in Asia. As shown in Figure S5, SDS-PAGE, WB, and TEM results indicated that we successfully obtained the HuNoV VLPs above. In the developed colorimetric assay, all non-GII.4 HuNoV VLPs yielded A620/A520 values below 0.4, indicating that these genotypes could not effectively compete with AuNPs for binding to the AP4-11 aptamer (Figure 5B).

### 3.8. Detection of HuNoV in Clinical Fecal Samples

**Figure 6:**
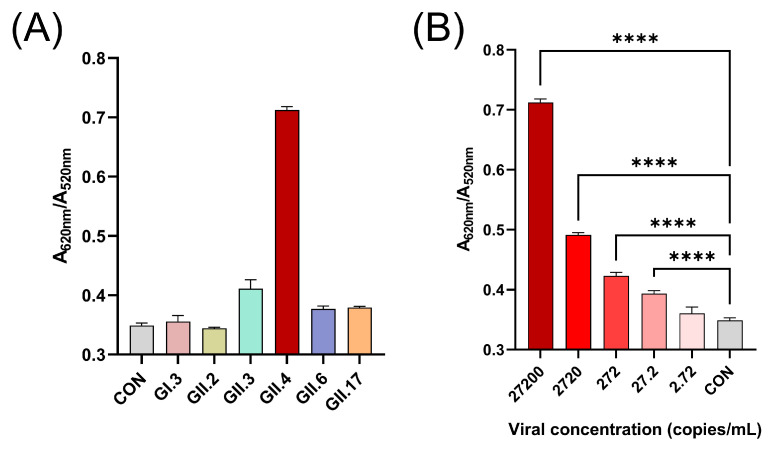
(**A**) A620/A520 values obtained by applying the colorimetric assay to clinical samples of different genotypes of HuNoV. (**B**) Response of the colorimetric assay to 10-fold serial dilutions of HuNoV GII.4 clinical samples. The data are reported as means ± SEMs. ****, *p* < 0.0001.

RNA extracted from HuNoV-positive fecal supernatants was quantified by RT-qPCR using a commercial kit. The viral concentration in the original supernatants ranged from 2.72 × 10^5^ to 119.69 × 10^5^ copies/mL. To minimize interference from complex fecal matrix components, all samples were diluted with ddH_2_O to a uniform concentration of 2.72 × 10^4^ copies/mL. As shown in Figure 6A, only the GII.4 HuNoV sample produced a distinct colorimetric signal, consistent with the results obtained using purified VLPs. Further colorimetric testing of serially diluted GII.4 HuNoV samples (27,200, 2720, 272, 27.2, and 2.72 copies/mL, respectively) revealed that the colorimetric assay could reliably distinguish samples with viral concentrations as low as 27.2 copies/mL from the negative control (Figure 6B). In summary, the developed colorimetric method enables effective and sensitive detection of GII.4 HuNoV in clinical fecal samples.

We also performed a comparative analysis between the colorimetric assay and RT-qPCR using a cohort of 24 clinical fecal samples, which included 12 RT-qPCR-confirmed GII.4-positive and 12 confirmed negative samples. The colorimetric method correctly identified all 12 positive samples, and 11 of the 12 negative samples were correctly classified as negative, resulting in only 1 false positive out of 24 samples. This preliminary comparison demonstrates a high level of concordance between the two methods. Future studies involving larger sample sets will be conducted to enable a more comprehensive statistical evaluation of the agreement between the two methods.

### 3.9. Recovery and Stability of the Colorimetric Assay

We first evaluated the impact of fecal matrix effects on colorimetric assay. As shown in Figure S6, at low dilution factors (1-, 10-, and 50-fold), the colorimetric signal was significantly compromised. The high concentration of fecal impurities, such as proteins, polysaccharides, and bile salts, likely induced non-specific aggregation of the AuNPs, leading to a high background signal, making it difficult to reliably distinguish between positive and negative samples. Meanwhile, a 100-fold dilution effectively mitigated these matrix effects. At this dilution, the concentration of interfering substances is reduced to a level that cannot lead to non-specific AuNP aggregation. This finding is consistent with how other studies handle complex samples [11,38].

To evaluate the feasibility and practical applicability of the developed aptasensor, recovery experiments were conducted by spiking various concentrations of GII.4 HuNoV VLPs into 100-fold diluted HuNoV-negative fecal samples. As summarized in Table 1, the recovery rates ranged from 91.74% to 106.43%, with relative standard deviations (RSD) of 0.46–6.70%, indicating satisfactory accuracy and reproducibility. These results demonstrate that the colorimetric assay is reliable and suitable for the detection of GII.4 HuNoV in complex fecal matrices.

Next, we investigated the effect of different storage conditions on the stability of aptamer-modified AuNP conjugates. As shown in Figure S7, the A620/A520 ratio for conjugates stored at 4 °C remained essentially unchanged for up to 14 days, indicating excellent colloidal stability without significant non-specific aggregation. Conjugates stored at room temperature showed a slight decrease in the ratio after 14 days, suggesting that 4 °C is the recommended storage condition.

A comparative analysis of our aptamer-based colorimetric assay against established norovirus detection methods is summarized in Table 2, highlighting its competitive performance. Our method demonstrates exceptional sensitivity among colorimetric platforms, with a detection limit of 27.2 copies/mL, which is significantly lower than other colorimetric approaches [39,40] and rivals that of more complex electrochemical sensors [3]. Crucially, our assay achieves an optimal balance between speed and sensitivity. It substantially outperforms the gold standard RT-qPCR [41] in assay time while maintaining high sensitivity. Furthermore, the use of a highly stable aptamer as the bioreceptor provides advantages in cost and robustness over antibody-based systems. This combination of high sensitivity, rapid results, and operational simplicity positions our method as a highly promising tool for practical norovirus detection. Another study [42] similar to ours also employed aptamers with gold nanoparticles and achieved comparable detection ranges in fecal samples. However, that study did not investigate the range of norovirus genotypes that could be detected.

### 3.10. Simulated Docking and Validation of AP4-11 with HuNoV VP1

**Figure 7:**
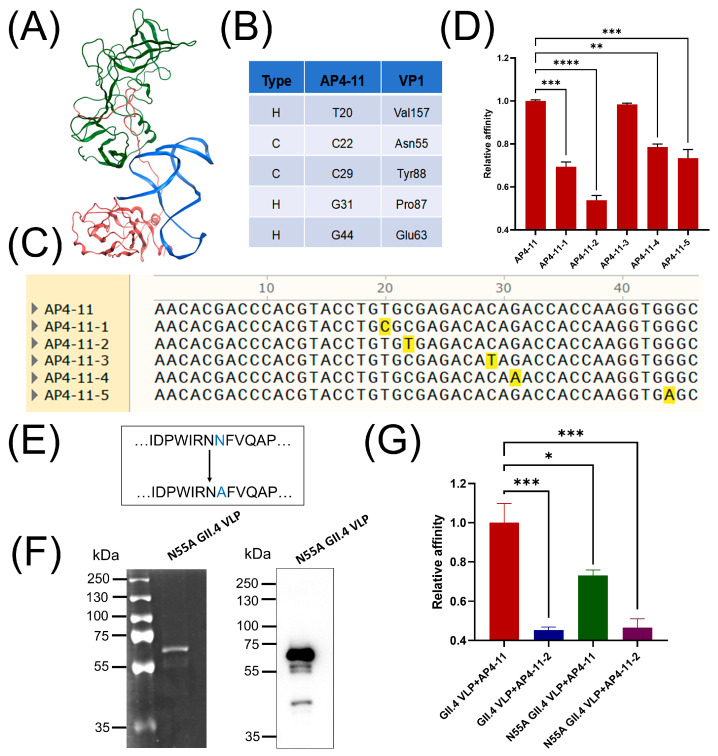
(**A**) Molecular docking results of AP4-11 with GII.4 HuNoV VP1: blue for aptamer AP4-11, red for the S domain, and green for the P domain of GII.4 HuNoV VP1. (**B**) Possible interaction sites of AP4-11 with GII.4 HuNoV VP1 obtained by MOE software. In the type, H denotes hydrogen, and C denotes covalent bonding. (**C**) Original and point-mutated sequences of AP4-11. Orange color indicates mutated base. (**D**) Comparison of the affinities of AP4-11 and mutated aptamers for GII.4 HuNoV VLPs. (**E**) Schematic representation of the amino acid mutation in GII.4 HuNoV VP1. (**F**) SDS-PAGE (**left**) and WB (**right**) of mutated GII.4 HuNoV VLPs. (**G**) Comparison of the affinity of primitive/mutant AP4-11 and primitive/mutant GII.4 HuNoV VLPs. The data are reported as means ± SEMs. ****, *p* < 0.0001, ***, *p* < 0.001, **, *p* < 0.01, and *, *p* < 0.05.

To elucidate the binding mechanism between AP4-11 and GII.4 HuNoV VLPs, we conducted structural prediction and molecular docking simulations of AP4-11 with GII.4 HuNoV VP1 (the major capsid protein of norovirus and the monomer of VLPs). The results indicated that AP4-11 binds to the S domain of GII.4 HuNoV VP1 (Figure 7A). As shown in Figure 7B, the predicted binding sites and interaction forces were all located within the stem–loop regions of AP4-11. We then introduced single-base mutations into AP4-11, generating five mutant aptamers (Figure 7C). ELASA analysis revealed that the binding affinity of AP4-11-1 (T20C), AP4-11-4 (G31A), and AP4-11-5 (G44A) for GII.4 HuNoV VLPs decreased by 22–31% compared to the original AP4-11. The most significant reduction was observed for AP4-11-2 (C22T), where a single-base substitution led to a 46% loss of binding affinity. In contrast, the affinity of AP4-11-3 (C29T) remained unchanged (Figure 7D).

The secondary structure of an aptamer, particularly its stem–loop motifs, is well established as a critical determinant for target binding [47,48]. Our secondary structure predictions for the five mutant aptamers (Figure S8) align with this. With the exception of AP4-11-3, all mutants exhibited notable structural alterations in these key stem–loop regions compared to the original AP4-11, explaining the marked decrease in binding affinity caused by single-base substitutions. A circular dichroism (CD) test was employed to investigate the structural changes of the aptamer AP4-11 upon binding to GII.4 HuNoV VLPs. Consistent with previous research, AP4-11 display distinct peaks at approximately 240 nm (negative peak) and 270 nm (positive peak), which are characteristic of B-type DNA, suggesting a stem–loop configuration in its secondary structure [49]. When binding to GII.4 HuNoV VLPs, significant decreases in both the positive and negative peak intensity were observed (Figure S9). A signal intensity decrease without a shift in peak position is a classic indicator of a structure being stabilized and becoming more rigid while retaining its fundamental secondary stem–loop structure [50]. This implies that the binding event likely induces locking of the aptamer’s flexible stem–loop into a more fixed and stable conformation, which is crucial for forming a specific and high-affinity complex with the VLP. The CD results confirm not only the binding between AP4-11 and GII.4 HuNoV VLPs but also elucidate that the binding mechanism involves a structural stabilization of the aptamer’s inherent stem–loop structure, which may be essential for its functional recognition.

Additionally, we expressed a mutated GII.4 HuNoV VLP (N55A) (Figure 7E) and validated it using SDS-PAGE and WB (Figure 7F). As shown in Figure 7G, mutations in either the aptamer or GII.4 HuNoV VLP led to reduced binding affinity, with aptamer mutations exerting a stronger effect than GII.4 HuNoV VLP mutations. These results were further corroborated by bio-layer interferometry measurements, which yielded consistent trends (Figure S10). AP4-11 exhibited the fastest binding kinetics and a nanomolar-level binding constant (K_D_ = 2.28 ± 0.03 nM, R^2^ = 0.98). Mutation in the GII.4 HuNoV VLP weakened the interaction, resulting in increased K_D_ values (K_D_ = 14.8 ± 0.16 nM, R^2^ = 0.96). And mutation in the aptamer led to lower binding rates and poorer fit quality (K_D_ = 4.62 ± 0.06 nM, R^2^ = 0.86). Similarly, the impact of aptamer mutation was greater than that of GII.4 HuNoV VLP mutation.

This observation may be attributed to the fact that a single-base mutation in the aptamer can induce significant conformational changes, disrupting not only the specific base–amino acid interaction but also other stabilizing contacts. In contrast, a single amino acid substitution in GII.4 HuNoV VLPs does not interfere with VLP assembly and likely affects only the local interaction at the mutated residue.

## 4. Conclusions

In summary, we have developed a colorimetric assay for the specific detection of GII.4 HuNoV using the optimized aptamer AP4-11 and AuNPs. The biotin-labeled aptamer was shown to stably bind to the AuNP surface, effectively preventing nanoparticle aggregation in NaCl solution. The assay exhibited high specificity toward GII.4 HuNoV and demonstrated a linear response to VLP concentrations ranging from 0.1 to 3.0 μg/mL, along with satisfactory recovery rates of 91.74–106.43%. When applied to clinical fecal samples, the method successfully detected GII.4 HuNoV at concentrations as low as 27.2 copies/mL. Furthermore, through a combination of structural prediction and experimental validation, we identified key bases in the aptamer and critical amino acid residues in GII.4 HuNoV VP1 that contribute significantly to the binding interaction. These findings support the potential of this assay for large-scale, rapid screening of GII.4 HuNoV in clinical settings.

## Figures and Tables

**Table 1:** Detection of the HuNoV-negative fecal sample spiked with different concentrations of GII.4 HuNoV VLPs using the developed aptasensor.

Sample	Spiked (μg/mL)	Found (μg/mL)	Mean Recovery (%)	RSD (%, n = 3)
1	1.0	0.96	95.72	6.70
2	2.0	2.13	106.43	9.03
3	2.5	2.43	97.31	0.46
4	3.0	2.75	91.74	2.38

**Table 2:** Performance of reported diagnostic methods for norovirus detection.

Detection	Bioreceptor	Assay Time (min)	Detection Limit	Reference
Colorimetry	Antibody	200	4.02 × 10^6^ copies/mL	[40]
Colorimetry	scFv	45	3 × 10^5^ copies/mL	[39]
Colorimetry	Aptamer	75	27.2 copies/mL	This work
Electrochemical	Antibody	–	121 copies/mL	[43]
Fluorescence	Antibody	<5	1.56 × 10^4^ copies/mL	[44]
SPR	Antibody	<5	96 copies/mL	[45]
RT-RPA-CRISPR/Cas12a	–	40	6.95 × 10^2^ copies/mL	[46]
RT-qPCR	–	360	10–100 pfu	[41]

scFv: single-chain variable fragments; SPR: surface plasmon resonance; RPA: recombinase polymerase amplification.

## Data Availability

Data are contained within the article and Supplementary Materials.

## Supplementary Materials

The following supporting information can be downloaded at: https://www.mdpi.com/article/10.3390/bios15110713/s1, Figure S1. Secondary structure of aptamers (A) AP4-9, (B) AP4-10, (C) AP4-12 and (D) AP4-13. Figure S2. UV-Vis spectra and color change of AuNPs (A) without and (C) with NaCl. TEM images of AuNPs (B) without and (D) with NaCl. Figure S3. Optimizations of colorimetric conditions (NaCl concentration). Relationship between absorbance and different concentrations of NaCl. Figure S4. A620/A520 value of the colorimetric reaction over time. The concentration of HuNoV GII.4 VLP detected was 5 μg/mL. Figure S5. (A) SDS-PAGE, (B) WB and (C) TEM of a series of genotypes of HuNoV VLPs. Figure S6. Detection performance of aptasensor in fecal samples with different dilution fold. Figure S7. Stability of aptamer-modified AuNP conjugates. Figure S8. Prediction of the secondary structure of (A) AP4-11, (B) AP4-11-1, (C) AP4-11-2, (D) 4-11-3, (E) AP4-11-4 and (F) 4-11-5. Bases in red boxes indicate mutated base. Figure S9. The circular dichroism spectrum of the 10.0 μM AP4-11 with/without 500 ng GII.4 HuNoV VLP. The data were collected at room temperature. Figure S10. Binding and dissociation curves and KD values of (A) AP4-11 with GII.4 VLP, (B) AP4-11-2 with GII.4 VLP, (C) AP4-11 with N55A GII.4 VLP and (D) AP4-11-2 with N55A GII.4. Table S1. Targets used in this study and their descriptions. Table S2. Aptamers modified from previously published aptamers for GII.4 HuNoV VLP.

## Abbreviations

The following abbreviations are used in this manuscript:

HuNoV	Human Norovirus
AuNPs	Gold Nanoparticles
VLP	Virus-Like Particle
PT-qPCR	Real-time Reverse Transcription Quantitative Polymerase Chain Reaction
SELEX	Systematic Evolution of Ligands by Exponential Enrichment
WB	Western blotting
TEM	Transmission Electron Microscopy
ELASA	Enzyme-Linked Aptamer Sorbent Assay
SDS-PAGE	Sodium Dodecyl Sulfate Polyacrylamide Gel Electrophoresis
RSD	Relative Standard Deviation
CD	Circular Dichroism

## References

[1] Kapikian A.Z., Wyatt R.G., Dolin R., Thornhill T.S., Kalica A.R., Chanock R.M. (1972). Visualization by Immune Electron-Microscopy of a 27-Nm Particle Associated with Acute Infectious Nonbacterial Gastroenteritis. J. Virol..

[2] Atmar R.L., Opekun A.R., Gilger M.A., Estes M.K., Crawford S.E., Neill F.H., Ramani S., Hill H., Ferreira J., Graham D.Y. (2014). Determination of the 50% human infectious dose for Norwalk virus. J. Infect. Dis..

[3] Kotwal G., Cannon J.L. (2014). Environmental persistence and transfer of enteric viruses. Curr. Opin. Virol..

[4] Bartsch S.M., Lopman B.A., Ozawa S., Hall A.J., Lee B.Y. (2016). Global Economic Burden of Norovirus Gastroenteritis. PLoS ONE.

[5] Teunis P.F.M., Moe C.L., Liu P., Miller S.E., Lindesmith L., Baric R.S., Le Pendu J., Calderon R.L. (2008). Norwalk virus: How infectious is it?. J. Med. Virol..

[6] Cannon J.L., Barclay L., Collins N.R., Wikswo M.E., Castro C.J., Magaña L.C., Gregoricus N., Marine R.L., Chhabra P., Vinjé J. (2017). Genetic and Epidemiologic Trends of Norovirus Outbreaks in the United States from 2013 to 2016 Demonstrated Emergence of Novel GII. 4 Recombinant Viruses. J. Clin. Microbiol..

[7] Costantini V., Grenz L., Fritzinger A., Lewis D., Biggs C., Hale A., Vinjé J. (2010). Diagnostic Accuracy and Analytical Sensitivity of IDEIA Norovirus Assay for Routine Screening of Human Norovirus. J. Clin. Microbiol..

[8] Tan X.H., Dey S.K., Telmer C., Zhang X.L., Armitage B.A., Bruchez M.P. (2014). Aptamers Act as Activators for the Thrombin Mediated-Hydrolysis of Peptide Substrates. ChemBioChem.

[9] Tuerk C., Gold L. (1990). Systematic Evolution of Ligands by Exponential Enrichment—Rna Ligands to Bacteriophage-T4 DNA-Polymerase. Science.

[10] Bunka D.H.J., Platonova O., Stockley P.G. (2010). Development of aptamer therapeutics. Curr. Opin. Pharmacol..

[11] Luan Y.X., Chen J.Y., Xie G., Li C., Ping H., Ma Z.H., Lu A.X. (2015). Visual and microplate detection of aflatoxin B2 based on NaCl-induced aggregation of aptamer-modified gold nanoparticles. Microchim. Acta.

[12] Liu Z.C., Zhang Y.F., Xie Y., Sun Y., Bi K.W., Cui Z., Zhao L.J., Fan W.F. (2017). An Aptamer-based Colorimetric Sensor for Streptomycin and Its Application in Food Inspection. Chem. Res. Chin. Univ..

[13] Goux E., Dausse E., Guieu V., Azéma L., Durand G., Henry M., Choisnard L., Toulmé J.J., Ravelet C., Peyrin E. (2017). A colorimetric nanosensor based on a selective target-responsive aptamer kissing complex. Nanoscale.

[14] Hu X.R., Chang K.K., Wang S., Sun X.Q., Hu J.D., Jiang M. (2018). Aptamer-functionalized AuNPs for the high- sensitivity colorimetric detection of melamine in milk samples. PLoS ONE.

[15] Gao H., Tian Y.L., Zhang M., Liu J.H., Yuan Y.W., Tan J.X., Ma A.J. (2020). Selection, Identification, and Application of Aptamers against Lectin to Establish an Aptamer-AuNPs Colorimetric Method for Detection of ABL. J. Food Qual..

[16] Rao X.Y., Zhang J.J., Cui J., Hu Y., Liu T., Chai J.F., Cheng G.F., He P.G., Fang Y.Z. (2013). Au nanoparticle-DNAzyme dual catalyst system for sensitively colorimetric detection of thrombin. Chem. Res. Chin. Univ..

[17] Anker J.N., Hall W.P., Lyandres O., Shah N.C., Zhao J., Van Duyne R.P. (2008). Biosensing with plasmonic nanosensors. Nat. Mater..

[18] Fried M.G., Crothers D.M. (1984). Kinetics and Mechanism in the Reaction of Gene Regulatory Proteins with DNA. J. Mol. Biol..

[19] Kozlov A.G., Lohman T.M. (1998). Calorimetric studies of *E. coli* SSB protein-single-stranded DNA interactions. Effects of monovalent salts on binding enthalpy. J. Mol. Biol..

[20] Samanta A., Medintz I.L. (2016). Nanoparticles and DNA—A powerful and growing functional combination in bionanotechnology. Nanoscale.

[21] Zhao J., Zhang Y.Y., Li H.T., Wen Y.Q., Fan X.Y., Lin F.B., Tan L.A., Yao S.Z. (2011). Ultrasensitive electrochemical aptasensor for thrombin based on the amplification of aptamer-AuNPs-HRP conjugates. Biosens. Bioelectron..

[22] Papavlassopoulos H., Mishra Y.K., Kaps S., Paulowicz I., Abdelaziz R., Elbahri M., Maser E., Adelung R., Röhl C. (2014). Toxicity of Functional Nano-Micro Zinc Oxide Tetrapods: Impact of Cell Culture Conditions, Cellular Age and Material Properties. PLoS ONE.

[23] Lee J.S., Ulmann P.A., Han M.S., Mirkin C.A. (2008). A DNA-gold nanoparticle-based colorimetric competition assay for the detection of cysteine. Nano Lett..

[24] Mun H., Jo E.J., Li T.H., Joung H.A., Hong D.G., Shim W.B., Jung C., Kim M.G. (2014). Homogeneous assay of target molecules based on chemiluminescence resonance energy transfer (CRET) using DNAzyme-linked aptamers. Biosens. Bioelectron..

[25] Pamies R., Cifre J.G.H., Espín V.F., Collado-González M., Baños F.G.D., de la Torre J.G. (2014). Aggregation behaviour of gold nanoparticles in saline aqueous media. J. Nanopart. Res..

[26] Zhou X.T., Wang L.M., Shen G.Q., Zhang D.W., Xie J.L., Mamut A., Huang W.W., Zhou S.S. (2018). Colorimetric determination of ofloxacin using unmodified aptamers and the aggregation of gold nanoparticles. Microchim. Acta.

[27] Ye H., Duan N., Gu H.J., Wang H.T., Wang Z.P. (2019). Fluorometric determination of lipopolysaccharides via changes of the graphene oxide-enhanced fluorescence polarization caused by truncated aptamers. Microchim. Acta.

[28] Sharifi S., Vahed S.Z., Ahmadian E., Dizaj S.M., Eftekhari A., Khalilov R., Ahmadi M., Hamidi-Asl E., Labib M. (2020). Detection of pathogenic bacteria via nanomaterials-modified aptasensors. Biosens. Bioelectron..

[29] Cheng C., Sun M.J., Li J.J., Xue Y.T., Cai X., Liu J., Wang X.L., Xu S.H., Xie Y.H., Zhang J.Q. (2025). Nucleic Acid Aptamers for Human Norovirus GII.4 and GII.17 Virus-like Particles (VLPs) Exhibit Specific Binding and Inhibit VLPs from Entering Cells. Int. J. Nanomed..

[30] Moe C.L., Sair A., Lindesmith L., Estes M.K., Jaykus L.A. (2004). Diagnosis of Norwalk virus infection by indirect enzyme immunoassay detection of salivary antibodies to recombinant Norwalk virus antigen. Clin. Diagn. Lab. Immunol..

[31] Zhou H.L., Chen L.N., Wang S.M., Tan M., Qiu C., Qiu T.Y., Wang X.Y. (2021). Prevalence and Evolution of Noroviruses between 1966 and 2019, Implications for Vaccine Design. Pathogens..

[32] Zuker M. (2003). Mfold web server for nucleic acid folding and hybridization prediction. Nucleic Acids Res..

[33] Liu X.M., Cao G.J., Ding H.M., Zhang D.J., Yang G., Liu N.L., Fan M., Shen B.F., Shao N.S. (2004). Screening of functional antidotes of RNA aptamers against bovine thrombin. FEBS Lett..

[34] Hermann T., Patel D.J. (2000). Biochemistry—Adaptive recognition by nucleic acid aptamers. Science..

[35] Shi M.H., Liu R.B., Zhang F.Y., Chitrakar B., Wang X.H. (2022). Screening of Single-Stranded DNA Aptamer Specific for Florfenicol and Application in Detection of Food Safety. Biosensors.

[36] Häkkinen H. (2012). The gold-sulfur interface at the nanoscale. Nat. Chem..

[37] Inkpen M.S., Liu Z.F., Li H.X., Campos L.M., Neaton J.B., Venkataraman L. (2019). Non-chemisorbed gold-sulfur binding prevails in self-assembled monolayers. Nat. Chem..

[38] Liu R., Zhang F., Sang Y., Liu M., Shi M., Wang X. (2022). Selection and Characterization of DNA Aptamers for Constructing Aptamer-AuNPs Colorimetric Method for Detection of AFM1. Foods.

[39] Martorell S., Santiso-Bellón C., Gozalbo-Rovira R., Quintero-Campos P., Luque D., Maquieira A., Rodríguez-Díaz J., Morais S. (2025). Synthetic biology-driven optoelectronic biosensor for rapid and highly sensitive norovirus detection in fecal samples. Biosens. Bioelectron..

[40] Ahmed S.R., Takemeura K., Li T.C., Kitamoto N., Tanaka T., Suzuki T., Park E.Y. (2017). Size-controlled preparation of peroxidase-like graphene-gold nanoparticle hybrids for the visible detection of norovirus-like particles. Biosens. Bioelectron..

[41] Hernández O.H., Gutiérrez-Escolano A.L., Cancio-Lonches C., Iturriaga M.H., Pacheco-Aguilar J.R., Morales-Rayas R., Arvizu-Medrano S.M. (2022). Multiplex PCR method for the detection of human norovirus, *Salmonella* spp., *Shigella* spp., and shiga toxin producing Escherichia coli in blackberry, coriander, lettuce and strawberry. Food Microbiol..

[42] Li X.Y., Zhao Y.Q., Gu W.C., Qian Y., Huang Q., Hu X.J., Xing H.B. (2024). A novel dual-mode aptasensor based colorimetry and electrochemical detection of norovirus in fecal sample. Anal. Biochem..

[43] Nasrin F., Khoris I.M., Chowdhury A.D., Boonyakida J., Park E.Y. (2022). Impedimetric biosensor of Norovirus with low variance using simple bioconjugation on conductive polymer-Au nanocomposite. Sensor Actuators B Chem..

[44] Gao J.S., Xue L., Li Y.J., Cai W.C., Miao S.D., Meng L.B., Ren S.L., Zhang J.M., Wang J., Wu S. (2024). Rapid and sensitive lateral flow biosensor for the detection of GII human norovirus based on immunofluorescent nanomagnetic microspheres. J. Med. Virol..

[45] Nasrin F., Chowdhury A.D., Takemura K., Lee J., Adegoke O., Deo V.K., Abe F., Suzuki T., Park E.Y. (2018). Single-step detection of norovirus tuning localized surface plasmon resonance-induced optical signal between gold nanoparticles and quantum dots. Biosens. Bioelectron..

[46] Qian W.D., Huang J., Wang X.F., Wang T., Li Y.D. (2021). CRISPR-Cas12a combined with reverse transcription recombinase polymerase amplification for sensitive and specific detection of human norovirus genotype GII.4. Virology.

[47] Wang L.L., Lee J.Y., Gao L.F., Yin J.K., Duan Y.K., Jimenez L.A., Adkins B., Ren W.D., Li L.H., Fang J. (2019). A DNA aptamer for binding and inhibition of DNA methyltransferase 1. Nucleic Acids Res..

[48] Xu G.H., Zhao J.J., Yu H., Wang C., Huang Y.Y., Zhao Q., Zhou X., Li C.G., Liu M.L. (2022). Structural Insights into the Mechanism of High-Affinity Binding of Ochratoxin A by a DNA Aptamer. J. Am. Chem. Soc..

[49] Wei L.K., Wang H.L., Wu J.M., Mao J., Wang S.J., Qiu J.Q. (2025). Ultra-sensitive label-free biosensor for doxorubicin detection by doxorubicin optimized aptamer. J. Food Compos. Anal..

[50] Xie Y., Yin J.W., Deng F., Xu L.F., Salminen K., Liu L.M. (2025). Sandwich-type electrochemical aptamer-based sensor for rapid nanomolar detection of anesthetic drug procaine in biofluids. Microchem. J..

