# Supplementary material for: Optimized Aptamer-Conjugated Gold Nanoparticles for Specific Detection of GII.4 Human Norovirus in Feces
Source: Biosensors (Basel). 2025 Oct 28;15(11):713. doi: 10.3390/bios15110713 (PMC12650235; doi:10.3390/bios15110713)
Supplement: Supplementary file 1 [file biosensors-15-00713-s001.zip › biosensors-3880233-supplementary.pdf]

SUPPLEMENTARY INFORMATION (SI)

Optimized aptamer-conjugated gold nanoparticles for specific detection of GII.4 human norovirus in feces

Materials and Methods

Optimization of NaCl concentration of the colorimetric assay

In order to determine the minimum NaCl concentration required for complete aggregation of AuNPs, 20 μL of NaCl solution in different concentrations (0, 100, 200, 500, 750, 1000, 1500 and 2000 mM) was mixed with 180 μL AuNPs respectively, and incubated at 37°C in dark. After 10 min, the color change was observed and measured over the wavelength ranging from 400 nm to 700 nm. In order to obtain more accurate NaCl concentration, a concentration gradient was set at 50 mmol/L intervals from 200 mM to 500 mM (200, 250, 300, 350, 400, 450 and 500 mM) and the same procedure was performed.

Kinetic analysis of the colorimetric assay

In order to monitor the aggregation degree of AuNPs after the addition of NaCl, colorimetric assay was performed with 5 μg/mL of HuNoV GII.4 VLP. The absorbance values at 520 nm and 620 nm were determined at different reaction time points (0 min, 2 min, 5 min, 5 min, 7 min, 10 min, 15 min, 20 min, 25 min, 30 min, 35 min, 40 min, 45 min, 50 min).

Circular dichroism (CD) parameters

The test was performed in 10 mM Tris-HCl (pH 7.4), 1.0 mM NaCl and 0.05 mM $MgCl_2$. The concentration of AP4-11 was 10.0 μM, and 500 ng GII.4 HuNoV VLP was added. The aptamer solution was maintained at 95 °C for 5 min, followed by cooling in ice water. The aptamer solution was incubated with the target overnight. The parameters for the CD test were as follows: wavelength range from 200 to 340 nm, the sensitivity, data space, scanning speed and bandwidth were about 5.0 mdeg, 0.2 nm, 50 nm/min and 1 nm, respectively.

Determination of dissociation constants

Binding affinities were determined by the BioLayer Interferometry (BLI) technique. GII.4 HuNoV VLP was first formulated into 150 nM, 75 nM, 37.5 nM and 18.75 nM solutions and candidate biotin-labelled aptamers were diluted to 100 nM with binding buffer (PBST+5 mM Mg^{2+}). Affinity assay experiments were then performed using the Molecular Interactor Fortebio Octet RED96 (USA), and the analysis steps were set up in the instrument software.

Table S1. Targets used in this study and their descriptions.

Target	Description	Experiments conducted
GII.4 HuNoV VLP	Virus-like particles of genotype GII.4 human norovirus	ELASA, establishment of colorimetric assay, specificity and recovery of colorimetric assay
GII.4 HuNoV	Fecal samples containing genotype GII.4 human norovirus	Detection efficiency of colorimetric assay in fecal samples
GII.4 HuNoV VP1	Major capsid protein of genotype GII.4 human norovirus	Simulated docking

Table S2. Aptamers modified from previously published aptamers for GII.4 HuNoV VLP.

Name	Sequence (5'-3')	Modified from [1]	ΔG (kcal/mol)
AP4-9	AACACGACGCACCTGTGTTGGACCCCACAGCCCATCAATGTGGGC	AP4-7+AP4-3	-12.39
AP4-10	AACACGACAACCCTGTGACCTCCCACAGCCCATCAATGTGGGC	AP4-2+AP4-3	-10.94
AP4-11	AACACGACCCACGTACCTGTGCGAGACACAGACCACCAAGGTGGGC	AP4-3+AP4-4	-15.49
AP4-12	AACAGACGAAACTGTGACTGTGCGAGACACAGACCACCAAGGTGGGC	AP4-1+AP4-4	-16.42
AP4-13	ATCACGACAACCCTGTGACATCACGACAACCCTGTGAC	AP4-2	-6.06

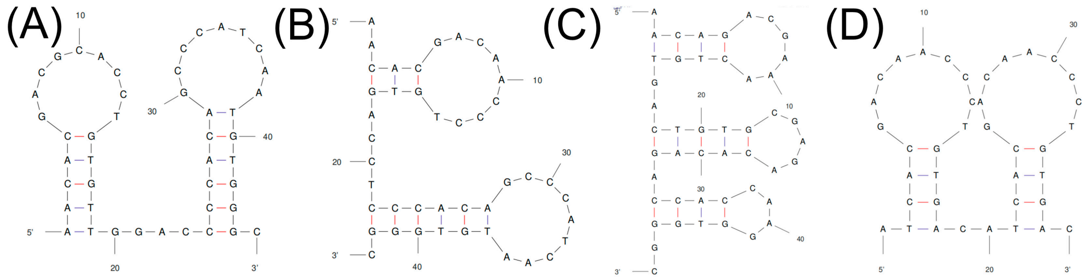

Figure S1. Secondary structure of aptamers **(A)** AP4-9, **(B)** AP4-10, **(C)** AP4-12 and **(D)** AP4-13.

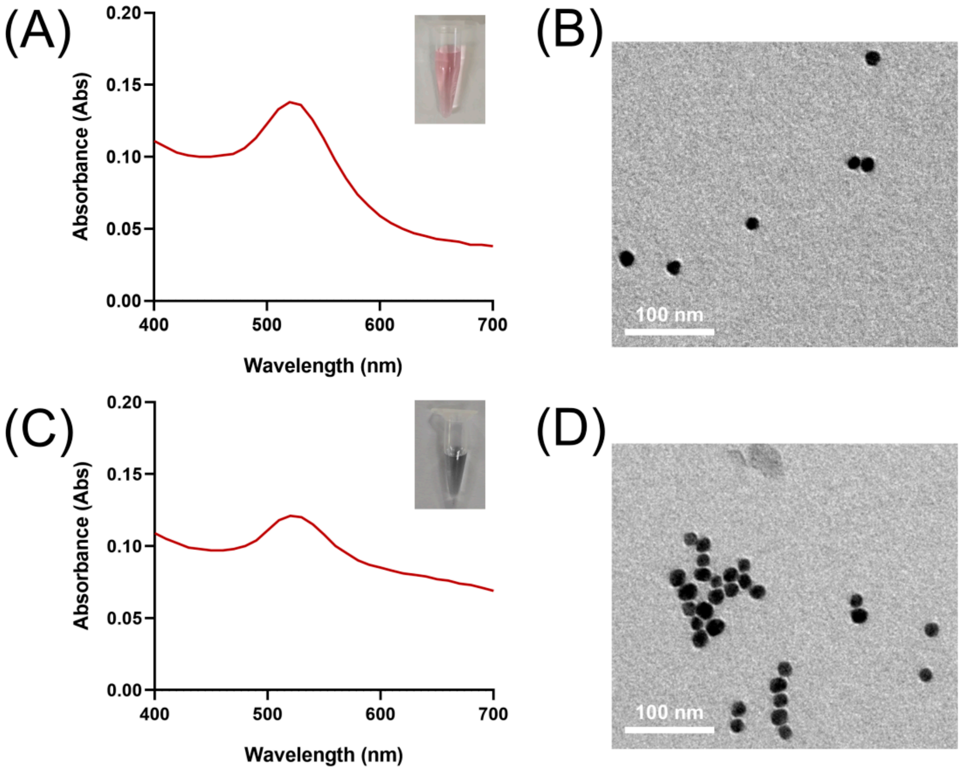

Figure S2. UV-Vis spectra and color change of AuNPs **(A)** without and **(C)** with NaCl. TEM images of AuNPs **(B)** without and **(D)** with NaCl.

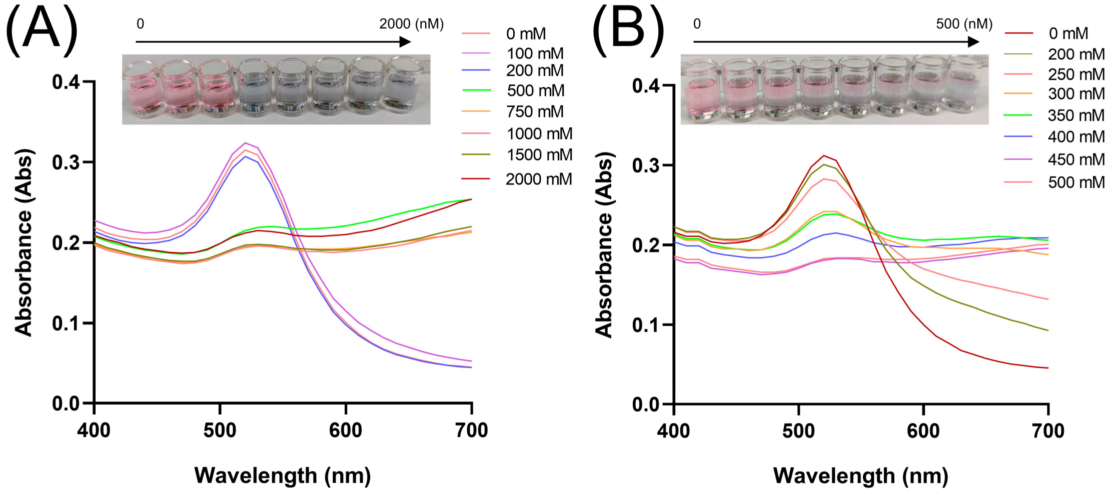

Figure S3. Optimizations of colorimetric conditions (NaCl concentration). Relationship between absorbance and different concentrations of NaCl.

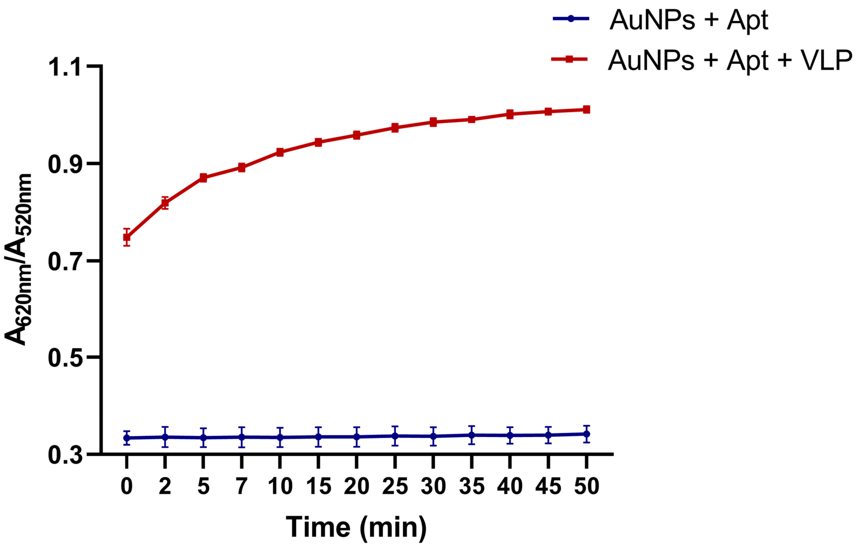

Figure S4. A620/A520 value of the colorimetric reaction over time. The concentration of HuNoV GII.4 VLP detected was 5 μg/mL.

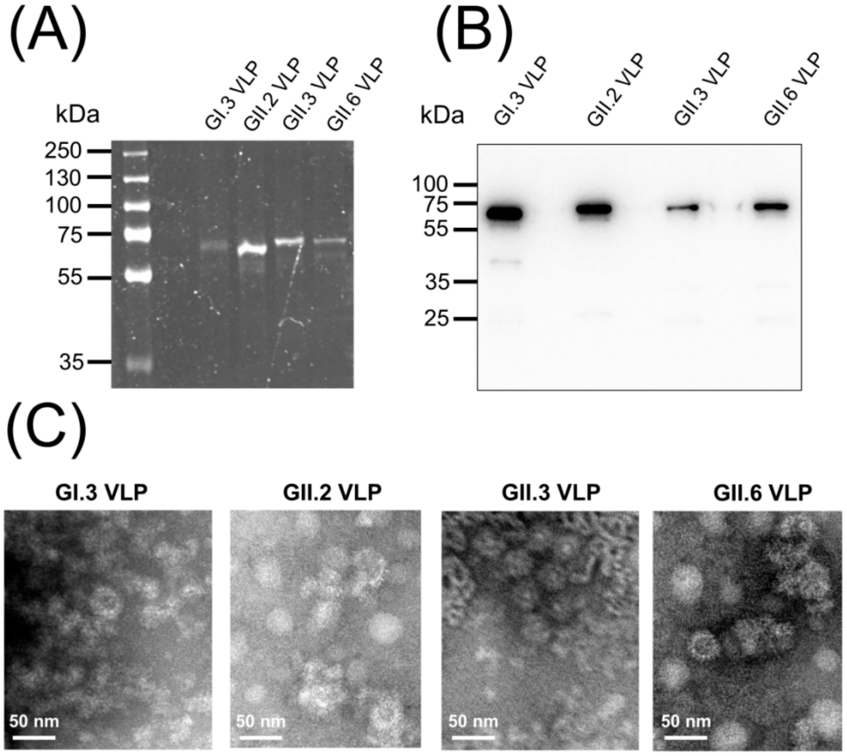

Figure S5. (A) SDS-PAGE, **(B)** WB and **(C)** TEM of a series of genotypes of HuNoV VLPs.

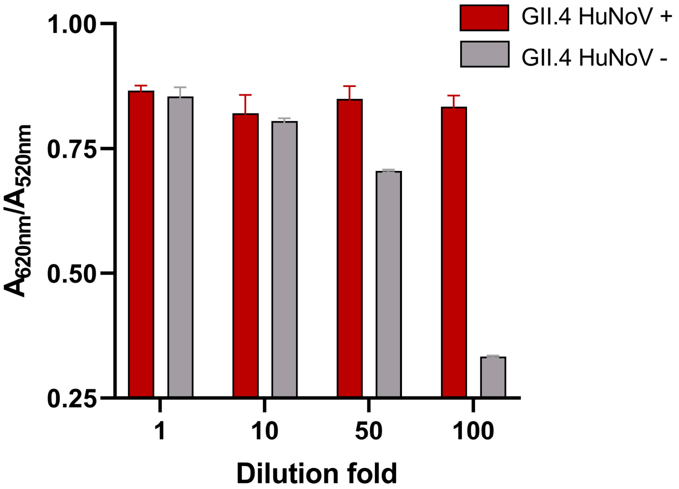

Figure S6. Detection performance of aptasensor in fecal samples with different dilution fold.

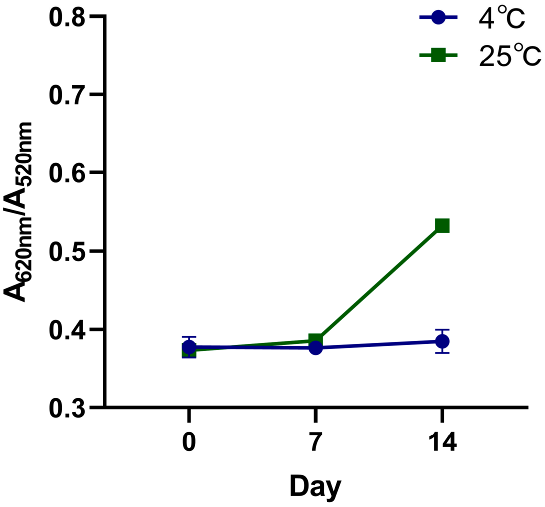

Figure S7. Stability of aptamer-modified AuNP conjugates.

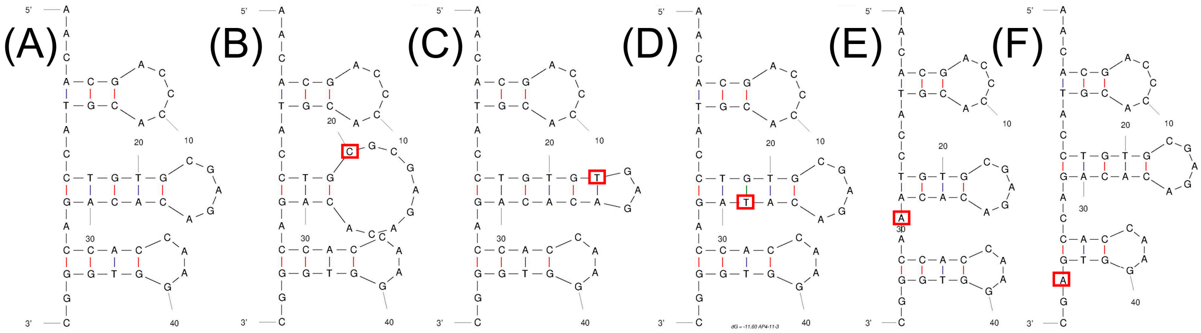

Figure S8. Prediction of the secondary structure of **(A)** AP4-11, **(B)** AP4-11-1, **(C)** AP4-11-2, **(D)** 4-11-3, **(E)** AP4-11-4 and **(F)** 4-11-5. Bases in red boxes indicate mutated base.

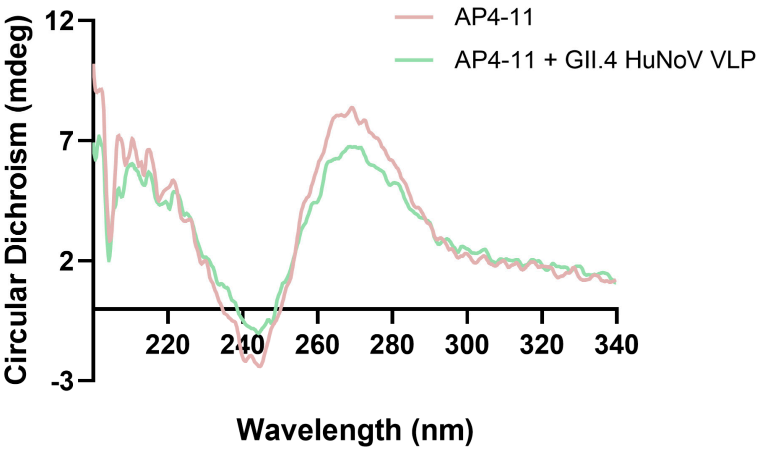

Figure S9. The circular dichroism spectrum of the 10.0 μM AP4-11 with/without 500 ng GII.4 HuNoV VLP. The data were collected at room temperature.

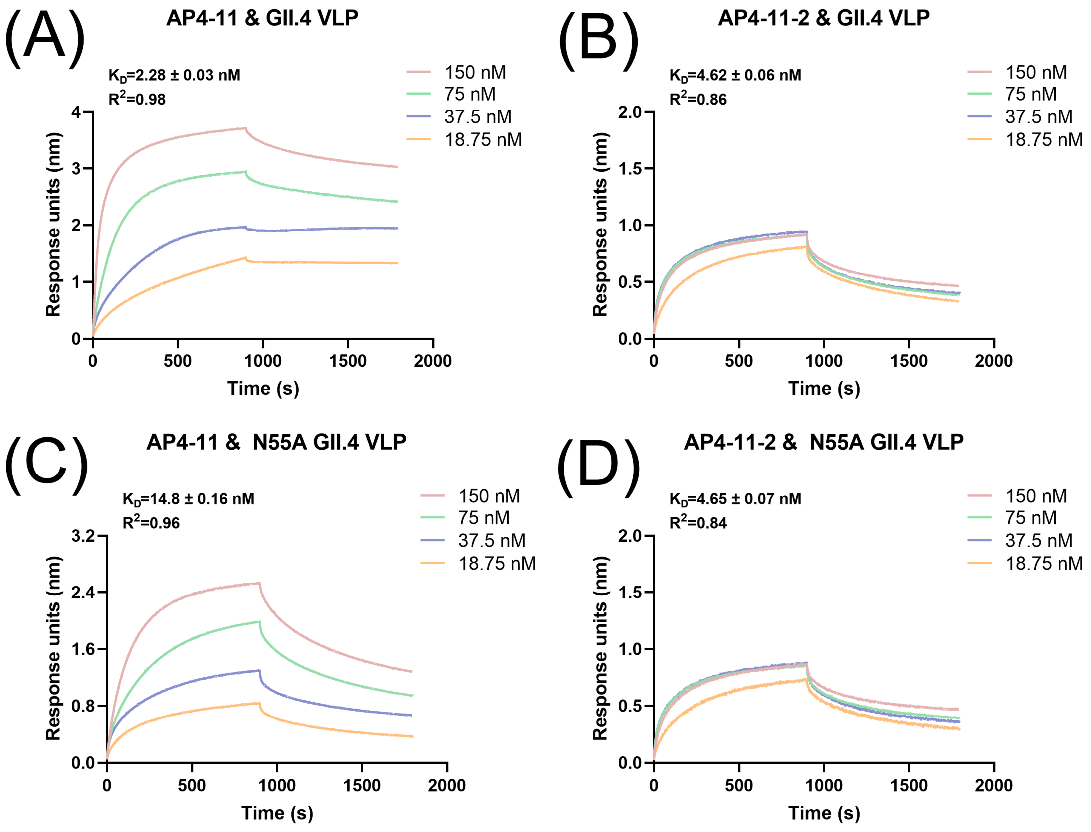

Figure S10. Binding and dissociation curves and K_D values of **(A)** AP4-11 with GII.4 VLP, **(B)** AP4-11-2 with GII.4 VLP, **(C)** AP4-11 with N55A GII.4 VLP and **(D)** AP4-11-2 with N55A GII.4.

1. Cheng C, Sun MJ, Li JJ, et al. Nucleic Acid Aptamers for Human Norovirus GII.4 and GII.17 Virus-Like Particles (VLPs) Exhibit Specific Binding and Inhibit VLPs from Entering Cells. *Int J Nanomed*. 2025;20(11):1789-1805. doi:https://doi.org/10.2147/Ijn.S495399
